# In Silico Investigation of a Surgical Interface for Remote Control of Modular Miniature Robots in Minimally Invasive Surgery

**DOI:** 10.1155/2014/307641

**Published:** 2014-09-09

**Authors:** Apollon Zygomalas, Konstantinos Giokas, Dimitrios Koutsouris

**Affiliations:** ^1^Life Science Informatics-Medical Informatics, Department of Surgery, University of Patras, Rio, 26500 Patras, Greece; ^2^Biomedical Engineering Laboratory, School of Electrical and Computer Engineering, National Technical University of Athens, 15780 Zografou, Athens, Greece

## Abstract

*Aim*. Modular mini-robots can be used in novel minimally invasive surgery techniques like natural orifice transluminal endoscopic surgery (NOTES) and laparoendoscopic single site (LESS) surgery. The control of these miniature assistants is complicated. The aim of this study is the in silico investigation of a remote controlling interface for modular miniature robots which can be used in minimally invasive surgery. *Methods*. The conceptual controlling system was developed, programmed, and simulated using professional robotics simulation software. Three different modes of control were programmed. The remote controlling surgical interface was virtually designed as a high scale representation of the respective modular mini-robot, therefore a modular controlling system itself. *Results*. With the proposed modular controlling system the user could easily identify the conformation of the modular mini-robot and adequately modify it as needed. The arrangement of each module was always known. The in silico investigation gave useful information regarding the controlling mode, the adequate speed of rearrangements, and the number of modules needed for efficient working tasks. *Conclusions*. The proposed conceptual model may promote the research and development of more sophisticated modular controlling systems. Modular surgical interfaces may improve the handling and the dexterity of modular miniature robots during minimally invasive procedures.

## 1. Introduction

Minimally invasive surgery is nowadays a consolidated alternative to the traditional open surgery for a number of operations. Minimally invasive surgical techniques include laparoscopy, single site surgery, and natural orifice transluminal endoscopic surgery. Laparoscopy has proved to be less traumatic for the patient, with minimal operative blood loss, less postoperative pain, accelerated recovery, and excellent cosmesis. A new promising minimally invasive approach is the laparoendoscopic single site (LESS) surgery, also known by a variety of other names (e.g., single incision laparoscopic surgery (SILS) and reduced port surgery (RPS)). LESS has become popular among surgeons as an alternative to standard laparoscopic surgery for a variety of operations [1]. The evolution of minimally invasive surgery to the natural orifice transluminal endoscopic surgery (NOTES) began in 2004 when Kalloo et al. published his study on the transgastric surgery [2]. NOTES is very fascinating in terms of surgical technique but its evolvement seems to be strictly connected to technology [3].

Informatics and robotics offer novel tools to the modern surgeon. The development of in vivo miniature robots for use in surgery is nowadays a reality with potential advantages and possible application in minimally invasive surgery in the future [4, 5]. A revolutionary idea is the development of modular miniature robots. Modular miniature robots are composed of small subunits (modules) which could be assembled and construct a functional mini-robot [6]. Controlling modular mini-robots is rather complicated. It is essential therefore to develop appropriate software and hardware technology that will provide the surgeon with all necessary information and give him an easy and precise control of his miniature assistants. Using robotic simulation software, we can virtually develop mini-robots and investigate their capabilities in silico.

The aim of this study is the experimental in silico investigation of a conceptual model of a surgical remote control interface for modular miniature robots that can be used in minimally invasive surgery.

## 2. Materials and Methods

**Figure 1:**
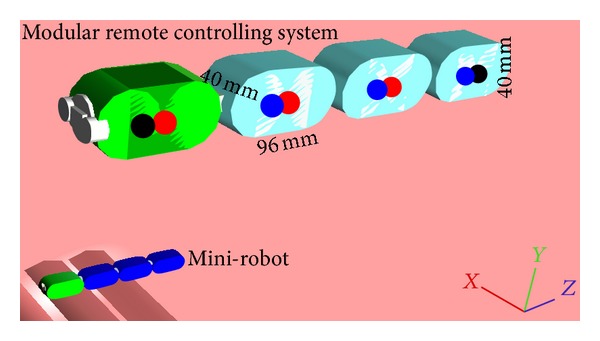
The modular remote controlling system (MCS) is identical to the modular miniature robot but in large scale, thus four times larger.

The development of our conceptual model is based on the idea that the user-surgeon could handle a modular remote controller similar, but in large scale, to the intra-abdominal modular mini-robot that he wants to control. He then could move the controller's modules as he tries to find a suitable configuration for his miniature assistant. We therefore designed a simple modular snake-like miniature robot consisting of four subunits and its respective modular remote controller and simulated them (Figure 1).

For the development and simulation of the controller and the respective mini-robot, we used the Webots version 6.0.0 (Cyberbotics, Switzerland) [7]. All modules were designed using simple 3D basic geometry objects. One cube and two cylinders construct the body of each module. The dimension of a mini-robot module is 24 mm × 10 mm × 10 mm. The modular remote controlling system (MCS) subunits have the same structure as those of the mini-robot but with dimensions of 96 mm × 40 mm × 40 mm, thus four times larger. This size should be rather handy for a surgeon.

**Figure 2:**
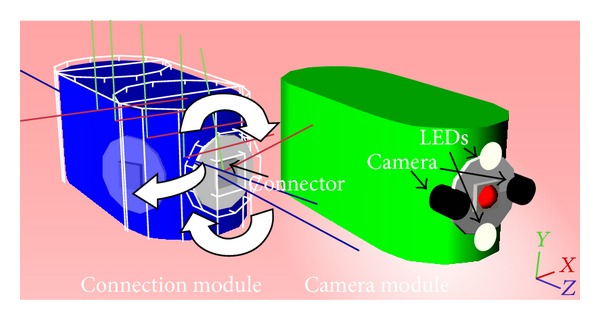
A connection module and a camera module. A servomotor gives motion on an arc of 180° on axis *Y*, (90° left and 90° right). A second motor gives a 360° motion on *Z* (or *X*) axis.

We designed two different types of modules: a connection module and a camera module (Figure 2). All modules are symmetrical in *Z* axis and are equipped with four active rotational servomotors which provide the assembled mini-robot with motion. Two servomotors are positioned on the front side and two on the rear side. In this way, one motor gives a 180° arc motion on axis *Y*, so as to have a motion of 90° left and 90° right and the other motor gives a 360° motion on axis *Z* (or *X*) for a complete rotation of the module when connected (Figure 2). Electromagnetic symmetrical connectors with controlled connection/disconnection are positioned on the front and rear sides of each subunit. The camera subunit of the mini-robot is equipped with two color cameras and two white light-emitting diodes (LEDs). The camera module of the controlling system does not have functioning cameras. Finally, each subunit is provided with an emitter and a receiver to achieve a wireless bidirectional module ID-based communication between the MCS and the mini-robot.

One red and one blue LED are mounted on each MCS subunit. These LEDs provide the user with visible information regarding the connection state. The blue LED is activated when the front connector is inside the magnetic field of the paired connector of another module and red LED is activated when the rear connector is inside the field. Detailed electromechanic robotic components were not designed. This was out of the aims of this study.

Three different controlling modes of the modular mini-robot were programmed. (A) The first is an absolute master-slave mapping mode where the configuration of the MCS is transmitted in real time to the modular mini-robot. For example, when the second module of the MCS is rotating, simultaneously the second module of the mini-robot performs an identical motion. (B) The second is a postaction mode (delayed master-slave mapping) in which the surgeon can move the modules of the MCS to achieve a preferred conformation and then transmit the new arrangement to the modular mini-robot when he desires (e.g., by pressing an appropriate button). (C) In the third mode, the user can select a preprogrammed simple motion or configuration from a list of actions. The MCS and the miniature robot execute simultaneously the command. Snake-like sinusoidal motions were programmed to achieve robot locomotion. Motion scaling was incorporated for accurate manipulation during the surgical procedure.

The C programming language with special libraries was used for the development of the simulation's programmable controllers using the built-in editor and compiler of Webots. All connection modules use a programmatically identical controller that positions the servomotors using a module ID-based control table. Camera modules use another controller which in addition implements the control of the video camera.

A number of physics parameters were defined using the physics nodes of Webots. Mass distribution, gravity force, and friction parameters were set, allowing the physics simulation engine to compute realistic forces.

**Figure 3:**
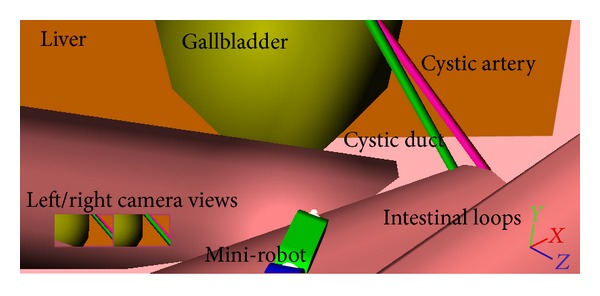
The intra-abdominal environment was simulated by simple 3D structures representing the intestine, the liver, and the gallbladder. On the lower left corner of the figure, the mini-robot's onboard camera views are shown. The gallbladder is suspended by a grasper like mini-robot (refer to [15]).

Intra-abdominal structures like the intestine, the liver, and the gallbladder were designed using simple 3D objects (Figure 3). The intraperitoneal environment was simulated in order to investigate vision and motion of the modular mini-robot in relationship to the controlling capabilities of the MCS.

Remote control was investigated regarding configuration, kinematics, coordination, localization, and user interaction.

## 3. Results

The modular controlling system allowed the user to immediately determine the conformation of the modular mini-robot. Real-time mode allowed for a standard use of the mini-robot executing pair commands as needed. This was the simplest way to control the miniature robot when it was stationary, thus while operating on tissues, for example. It seems to be impossible to provoke locomotion of the mini-robot using the real-time mode, as this procedure needs a quick and precise coordination of all the subunits. Fast changes of the conformation using rotational movements with over 0.25 rad/sec resulted in unpredictable positioning of the robot mainly due to moment of forces (Table 1). The actuation speed is user-dependent. An actuation speed limiter resolved the problem but restricted the user performance. It was easier to operate in real-time mode when the rear subunit of the system was fixed, thus a configuration similar to an external magnetic anchoring system (MAGS) [8]. It was difficult to control more than four subunits in real-time mode. The larger number of subunits complicated the behavior of the mini-robot because of additional forces like moment forces and friction which the user has to consider mentally in real-time.

The postaction mode helped to find different conformations without synchronous modification of the micro-robot. The user was able to find the most suitable configuration for his activity. However, in many occasions, using this mode significantly changed the arrangement and orientation of the intra-abdominal robot in an unpredictable way. This fact is caused by the torque which was developed during high speed (>0.25 rad/sec) rotational motions of the modules and subsequently due to collisions with some surrounding structures. For that reason, the postaction mode speed was set to 0.1 rads/sec. This mode proved useful when it was used to predict a stable configuration for operation. The postaction mode is by default ineffective for locomotion. It seems that there is no limit in the number of subunits that can be handled by this controlling mode, although the higher the number, the harder the control and the prediction of its result.

The preprogrammed action mode proved to be the only one to provide an acceptable locomotion of the mini-robot and a quick rearrangement of its subunits. During the execution of commands under this mode, the MCS was accessible to the operator. However, the operator could only start and stop a preprogrammed action and not modify it. There seems to be no limit in the number of subunits that can be handled using this mode. Regarding robot locomotion, the more the subunits used, the better the results were. High rotational speed (>0.25 rad/sec) of the servomotors gave acceptable locomotion of the robot. Nevertheless, if a preprogrammed conformation without locomotion was desirable, then the high rotational speed of the servomotors resulted in some occasions to unpredictable positioning of the robot mainly due to torque. A speed limit of 0.1 rad/sec was set for all the preprogrammed actions except for the sinusoidal locomotion.

**Figure 4:**
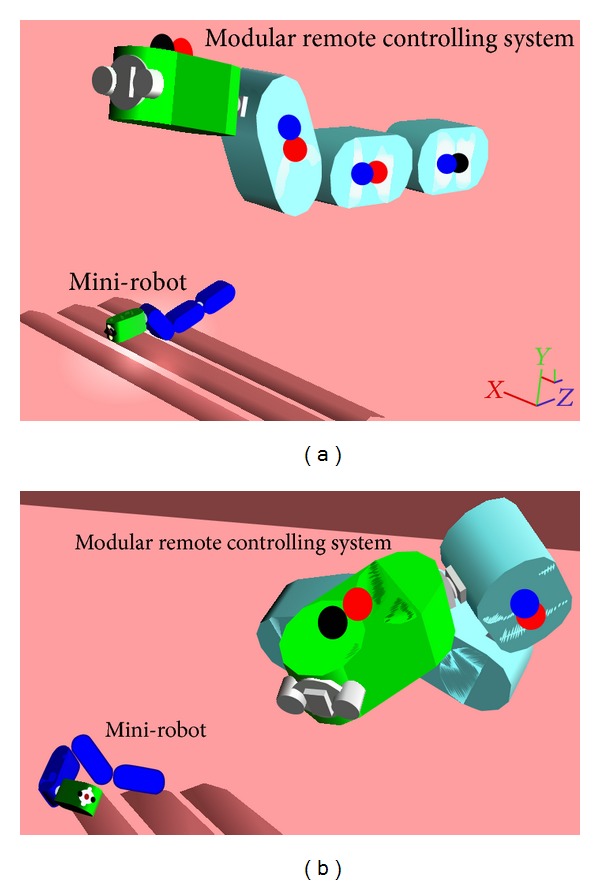
(a) Identical conformation between MCS and mini-robot with slightly different (acceptable) positioning of the second. (b) Identical conformation but very different orientation and positioning.

The configuration of the modular mini-robot was always identified only by observing the MCS conformation. However, its positioning and orientation were not predictable all the time. This principally occurred due to collisions with the surrounding structures or slippage (i.e., on intestine) (Figure 4). Another reason for the unpredictable positioning was the development of considerable torque during high speed rotational motions (>0.25 rad/sec). The mini-robot executed all the commands sent by the MCS but its modules motion depended on their interaction with the surrounding structures which in some occasions blocked a motion or even forced a disconnection of a module.

The LEDs of the MCS provided visual information regarding the connection state of the miniature modules. This proved to be useful while constructing the modular mini-robot. It was also helpful when accidental or voluntary disconnection occurred. All the commands sent by the MCS were logged in order to let the user read information regarding his actions.

## 4. Discussion

Surgery is historically connected with scars and pain. This aspect can be eliminated by minimally invasive surgery and especially with LESS and NOTES. However, software, hardware, novel surgical tools, and approaches should be evolved. Miniature robots can be equipped with surgical tools and sensors in order to provide information from the abdominal cavity and the possibility of remotely controlled surgical operations [9]. Miniature robots could be handled even by nonspecialized personnel and remote-controlled by surgeons miles away from the patient [10].

The use of modular mini-robots for minimally invasive surgery poses difficulties to surgeons related to the coordinative controlling of the robotic subunits. A single module by itself cannot operate, but modules arranged together can achieve complex tasks and controlling such a system could be rather difficult. The surgical robots are commonly controlled from outside the patient's body under indirect video assisted vision with the use of a joystick-like surgical interface [4, 11, 12]. Snake-like modular mini-robots can be in some cases compared to flexible endoscopes. Having this in mind, we should consider the study of Allemann et al. who proved that the use of robotized endoscopes with joystick interface is insufficient to enhance immediate intuitiveness of flexible endoscopy for NOTES [13]. Because of the complexity of locomotion and the precision of the task that modular mini-robots should perform, novel remote controlling systems should be developed in order to make the surgeon, and not the engineer, operate. The surgeon who will use modular miniature robots should feel safe and relaxed; therefore, a remote controlling system should be simple and accurate.

Wortman et al. presented a miniature robot prototype in which surgical interface is a kinematically matched master-slave configuration scaled model of the robot's arms [14]. This master system allows the surgeon to control the robot by directly mapping each joint. The scale of master-slave is 1.8 : 1 in length. Our conceptual model has the same basic idea of pairing master-slave configuration but is proposed for totally intracorporeal modular miniature robots (internal robots) [15]. The system of Wortman et al. provides the user with direct control over each joint of the mini-robot, allowing for a better sense of control. However, because none of the joints is provided with motors, the master arm cannot be held in place when the robot arm is locked. The master must be returned to an orientation similar to the robot before it can be unlocked. Our conceptual model of controller is equipped with motors and by default its configuration is always the same as that of the intra-abdominal miniature robot.

Our experimental investigation gave some useful information regarding the type of control, the working speed of the servomotors, and the number of modules that a modular robot can have in order to be efficiently controlled (Table 1). Although the real-time control of the mini-robot is the most natural to be utilized during a surgical operation, it is difficult to control more than four subunits and it is impossible to induce locomotion of the whole robotic system. On the other hand, the usefulness of postaction mode resulted in doubt. This situation was created because of the unpredictable interaction of the robotic subunits with the surrounding structures during rearrangement. Furthermore, if the rotational speed of the servomotors was high during the rearrangements, then the torque was considerable. This fact should be taken into consideration in such a miniature scale. However, using this type of control, it was easier to find an appropriate conformation for a stable operational robot. The preprogrammed mode was the only one that provided the robot with locomotion. However, when activated, the robot conformation changes as the preprogrammed commands need, and this sometimes may have unpredictable results regarding the interactions with the surrounding structures and the modules moment forces as mentioned above.

The positioning of the subunits on the modular mini-robot was always known and in the case of the real world the surgeon could virtually “touch” his mini-robot through his twin big brother remote controller. Slow and steady motion of the modules is desirable in order to minimize torque and achieve desirable conformation, positioning, and orientation. However, a more detailed study by a team of robotics specialists and the use of more sophisticated simulation libraries that take into account all the characteristics of the robot and the environment in which it operates are required. It is essential to construct and study in vivo such a mini-robot and its surgical interface. Effective cooperation between surgeons, robotics specialists, and informatics specialists is fundamental for a successful use of miniature robots in minimally invasive surgery.

Although this was a simple and basic simulation, it may be useful for a future construction of remote controlling surgical interfaces that can be used in order to control modular miniature robots during minimally invasive surgical procedures. Another idea is to modify this conceptual model as a “hand glove” which a surgeon can wear and control with it his miniature assistants (every module could be a phalange or a part of the upper limb, e.g., the front camera module paired to a finger, the second module paired to the hand, the third module paired to the forearm, and the fourth module paired to the arm). The present work represents only a conceptual in silico experimental study with no intention to solve mechanical, electrical, and robotic engineering problems in general.

## 5. Conclusions

The design of the proposed conceptual model may facilitate the development of more sophisticated and complex modular controlling systems. Modular surgical interfaces may improve the handling and the dexterity of modular miniature robots during minimally invasive procedures.

## Figures and Tables

**Table 1:** Modular remote controlling system operating modes.

Operating mode	Actuation speed	Ideal number of modules	Pros	Cons
Absolute master-slave mapping	User-dependent	4	Natural operation	User dependent action speed control
Delayed master-slave mapping	Set to 0.1 rad/sec	4–6	Predictable conformation	Unpredictable positioning and orientation
Preprogrammed	Set according to the desired action	4–6	Locomotion	No user interaction
